# Can selection for resistance to OsHV-1 infection modify susceptibility to *Vibrio aestuarianus* infection in *Crassostrea gigas*? First insights from experimental challenges using primary and successive exposures

**DOI:** 10.1186/s13567-015-0282-0

**Published:** 2015-12-09

**Authors:** Patrick Azéma, Marie-Agnès Travers, Julien De Lorgeril, Delphine Tourbiez, Lionel Dégremont

**Affiliations:** Ifremer, Laboratoire de Génétique et Pathologie des Mollusques Marins, Avenue Mus de Loup, 17390 La Tremblade, France; Ifremer, IHPE, UMR 5244, Univ. Perpignan Via Domitia, CNRS, Univ. Montpellier, 34095 Montpellier, France

## Abstract

Since 2008, the emergent virus OsHV-1µvar has provoked massive mortality events in *Crassostrea gigas* spat and juveniles in France. Since 2012, mortality driven by the pathogenic bacteria *Vibrio aestuarianus* has stricken market-sized adults. A hypothesis to explain the sudden increase in mortality observed in France since 2012 is that selective pressure due to recurrent viral infections could have led to a higher susceptibility of adults to *Vibrio* infection. In our study, two OsHV-1-resistant lines (AS and BS) and their respective controls (AC and BC) were experimentally challenged in the laboratory to determine their level of susceptibility to *V. aestuarianus* infection. At the juvenile stage, the selected lines exhibited lower mortality (14 and 33%) than the control lines (71 and 80%), suggesting dual-resistance to OsHV-1 and *V. aestuarianus* in *C. gigas*. Interestingly, this pattern was not observed at the adult stage, where higher mortality was detected for AS (68%) and BC (62%) than AC (39%) and BS (49%). These results were confirmed by the analysis of the expression of 31 immune-related genes in unchallenged oysters. Differential gene expression discriminated oysters according to their susceptibility to infection at both the juvenile and adult stages, suggesting that resistance to *V. aestuarianus* infection resulted in complex interactions between the genotype, stage of development and immunity status. Finally, survivors of the *V. aestuarianus* challenge at the juvenile stage still exhibited significant mortality at the adult stage during a second and third *V. aestuarianus* challenge, indicating that these survivors were not genetically resistant.

## Introduction

**Figure 1:**
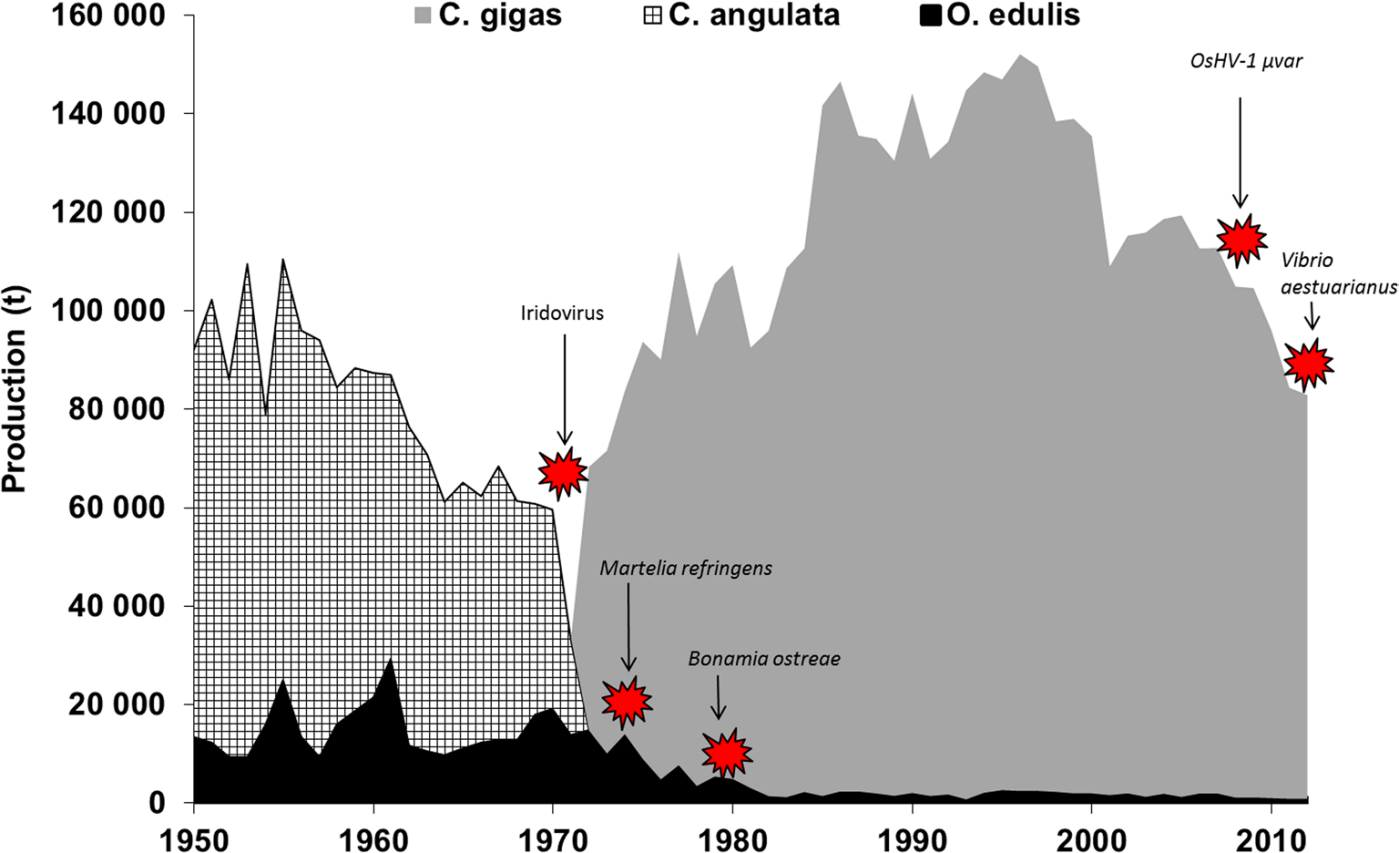
**French oyster production of** ***C. angulata***, ***C. gigas***,** and** ***O. edulis*** **since 1950.** The main diseases that affected the production are indicated with red stars.

The French oyster industry has regularly suffered from massive mortality episodes (Figure 1). In the early 1970s, the production of the Portuguese oyster *Crassostrea angulata* collapsed due to massive mortality related to an iridovirus [1], and the production of the flat oyster *Ostrea edulis* was significantly reduced due to two parasites (*Martelia refringens and Bonamia ostreae*) [2]. Once a disease affecting an oyster species has been introduced or detected in an area, resources to minimize its effect on oyster populations or production are very constrained. Neither large-scale drug use nor vaccination (because invertebrates have no acquired immunity) is possible in open marine areas due to the scale of the environment, and seawater or other organisms can easily convey pathogens from the reservoir to naïve stocks, thereby favoring the transmission of a disease. In this context, two main solutions have been proposed to sustain French oyster production: (1) develop a selective breeding program to enhance disease resistance using the genetic resources available in oyster populations, and more radically (2) introduce another species that is not sensitive to the disease. This second step was taken in France during the 1970s with the massive introduction of *Crassostrea gigas* from Japan and British Columbia to replace *C. angulata* during the RESOR operation (Figure 1) [3]. However, the introduction of new species is not recommended because it can lead to the introduction of new diseases in local populations [4], competition for habitats and resources, new invasive species and other constraints (regulatory rules, preliminary studies and biological barriers).

French oyster production of *C. gigas* has ranged from 100 000 to 150 000 tons for several decades, but has unfortunately begun to decrease due to two diseases (Figure 1). Indeed, massive mortality events have occurred every year since 2008, with high mortality rates for spat and juveniles (over 70%). A particular OsHV-1 genotype (µvar) that was first described during a period of *C. gigas* mortality in 2004–2005 in Normandy [5] has been ascribed to the mortality [6]. Moreover, significant mortality has been observed in market-sized adults since 2012 [7–9], and *C. gigas* production is expected to decrease again (Figure 1). The main pathogenic agent found in the dying oysters harvested during these mortality episodes belonged to the species *Vibrio aestuarianus*. Because it would not be reasonable to introduce another oyster species to replace C. gigas, the possibility of genetic selection for disease resistance might limit the impact of diseases on wild and cultivated oysters.

High mortality rates related to OsHV-1µvar have been observed in the field since 2008 [10], and it is probable that viral pressure on wild and cultivated oyster populations has been significant. Therefore, the emergence of high mortality in adults has made it legitimate to investigate whether the selection provoked by viral infection has an impact on the susceptibility of adults to bacterial infections and whether there are correlations or trade-offs between resistance to OsHV-1 and the expected resistance to *V. aestuarianus.* Consequently, it would be interesting to study whether the mass selection breeding program for *C. gigas* currently being managed at Ifremer [11] could enhance disease resistance to both OsHV-1 and *V. aestuarianus*.

Evidence of OsHV-1 resistance was demonstrated in spat *C. gigas* in 2009 using oysters selected based on their higher resistance to the summer mortality phenomenon in 2001 [12–14]. More recently, OsHV-1 resistance was found to be a highly heritable trait in *C. gigas* spat under field and laboratory conditions [11, 15]. However, experimental selective breeding programs focused on *V. aestuarianus* resistance have not been described to date, and a relationship between resistance to OsHV-1 infection and *V. aestuarianus* infection has not been reported.

One hypothesis to explain disease resistance could be linked to host defenses, such as the immune capacity. Previous works identified markers for oyster survival capacity [16–19]. These studies led to the identification of a set of genes whose expression was either up-regulated in oysters able to survive virulent Vibrio infection [16, 20] or differentially regulated in the hemocytes of oysters with a high capacity for survival [18].

The objective of this study was to investigate: (1) the resistance to *V. aestuarianus* infection in *C. gigas* at the juvenile and adult stages under laboratory-controlled conditions using two stocks of oysters and (2) to analyze the association of the survival capacity with the basal expression levels of a selection of immune-related genes. For each stock, a control line and a line selected for higher survival at the spat stage under field conditions (which was also related to higher resistance to OsHV-1 infection) were evaluated. First, experimental OsHV-1 infection was performed to confirm the level of resistance of each line to the viral infection. Then, two approaches were used to test for resistance to *V. aestuarianus* infection. The first approach challenged the oysters in primary exposures at the juvenile stage or the adult stage to determine the level of resistance according to the size and/or age of the oysters. The second approach used successive infections at the adult stage with the survivors of the previous experimental infections to determine whether the survivors became resistant to the bacterial infection. Finally, we evaluated the immune status of non-stimulated oysters before the onset of bacterial infection under laboratory conditions.

## Materials and methods

### Oysters

A mass selection to increase survival in *C. gigas* was performed in two stocks (named A and B) of wild oysters sampled from two sites in the Marennes-Oléron Bay (Charente Maritime, France) in 2008. For each line, a base population G_0_ was produced in 2009, and a sub-sample was kept in our facilities to avoid disease-related mortality and to produce the control line of the following generation (G_1_-C). This control allowed the assessment of the effects of changing environmental conditions during the course of the experiment to estimate the response to selection [11]. The other sub-sample of oysters was deployed in the field, where mortality outbreaks caused by OsHV-1 were routinely observed each year since 2009 [21]. Then, the survivors were spawned in 2010 to produce the selected line G_1_-S. The same approach was used in February 2011 and March 2012 to produce G_2_ and G_3,_ respectively. Four sub-lines were produced for the selected line from G_2_; further details are given in [11].

**Figure 2:**
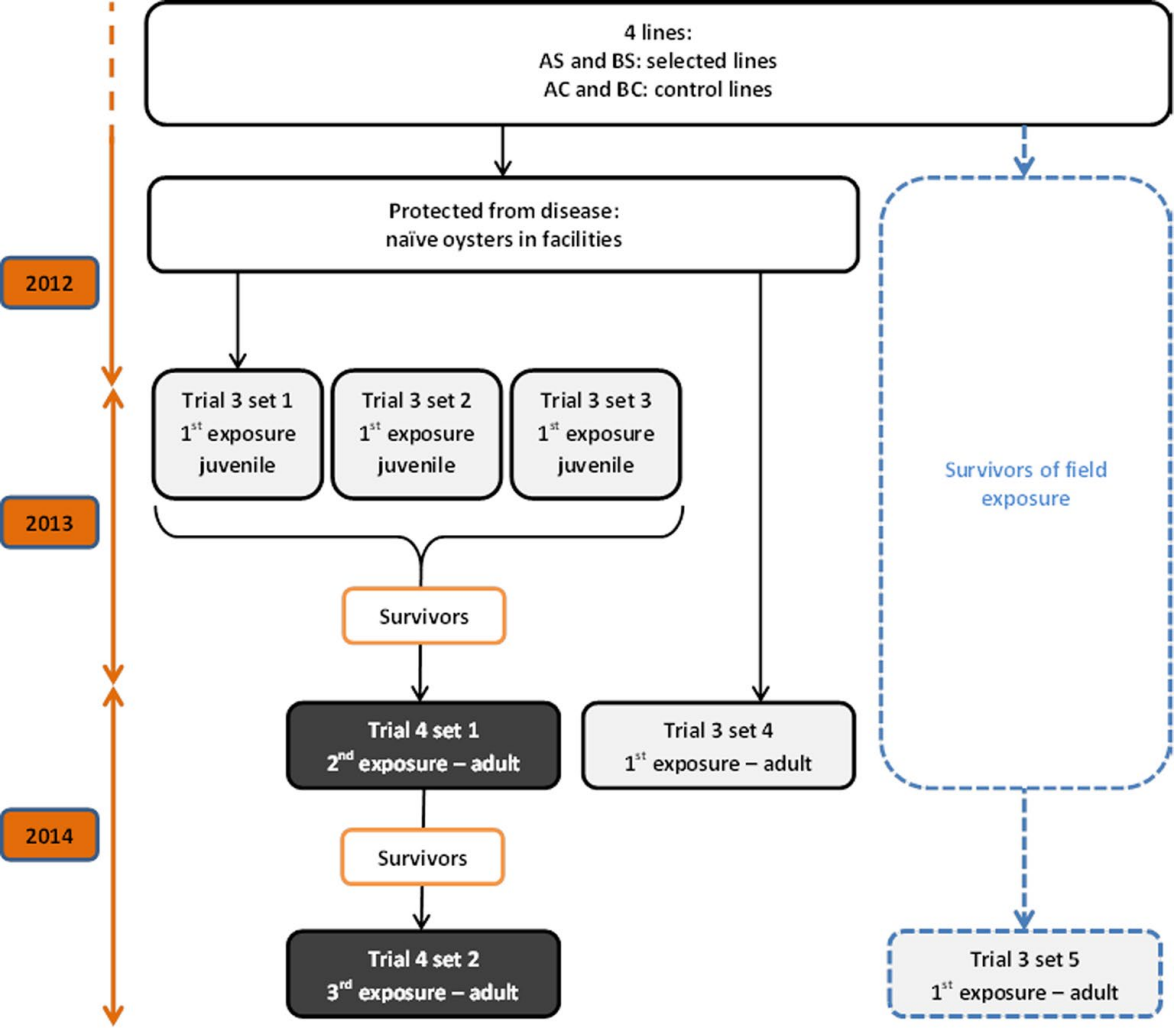
**Summary of the production and exposure to** ***V. aestuarianus*** **by cohabitation challenges.** Trials 3 and 4 were performed for the control and selected lines at either the juvenile or adult stages. Light grey boxes indicate primary-challenge and dark grey boxes indicate a second and third exposure to the bacteria.

The oysters used in this study were the control lines AC and BC of G_3_ and the selected lines AS and BS, which were the best sub-lines for survival and OsHV-1 resistance in the field. The field evaluation of the C and S lines at the spat stage during the summer of 2012 confirmed a higher mortality for the C lines (92.9%) compared with the S lines (32.0%). Nevertheless, the oysters used in our experimental infections were either kept in our inland facilities to avoid disease-related mortality or deployed to the field in October 2012 prior to their evaluation in the laboratory (Figure 2).

### Viral and bacterial suspensions

The viral suspension was obtained using the protocol of Schikorski [22]. Briefly, naïve and unselected hatchery-produced oysters were infected by injecting 50 µL of a previous viral suspension after “anesthesia”. Dead oysters were dissected; the mantle and gills were removed, pooled, diluted, crushed and filtered using a 0.22-µm filter to obtain a clarified tissue homogenate.

The *Vibrio* strain used in the bacterial challenges was the highly pathogenic strain 02/041 that was isolated during a mortality episode in adults. This strain was previously studied and was included in this study as a reference strain [23]. The *Vibrio* suspension was obtained from an isolate maintained at −80 °C. The bacterial strain was placed in liquid Zobell and incubated for 24 h at 20 °C with constant shaking at 20 rpm. The resulting solution was centrifuged at 3200 × *g* for 10 min. The supernatant was discarded, and the pellet was washed and suspended in sterile artificial sea water (SASW).

### Mortality induction protocols

Two types of experimental infection protocols were used to evaluate disease resistance in *C. gigas*: a by-injection protocol and a by-cohabitation protocol. For all trials, the seawater was filtered, UV treated and maintained at 21 °C with adequate aeration and without the addition of food. For the large volume tanks (150 L), a recirculating system was used to optimize the horizontal transmission of the disease. The salinity ranged between 29.5 and 36.7% for all trials.

For the by-injection protocol, pathogenic agents were directly injected into the adductor muscles of oysters to test their disease resistance. First, oysters were “anesthetized” in a solution containing magnesium chloride (MgCl_2_, 50 g/L) in a mixture of seawater and distilled water (1:4, v:v) for 4 h. Subsequently, 50 µL of the infectious solution (bacterial or viral suspension) was injected into the adductor muscle using a 1 mL micro-syringe equipped with an 18 g needle. The injected oysters were either naïve oysters of the selected or control lines or naïve unselected hatchery-produced oysters that were used as “sources” for the horizontal transmission of the disease to the selected and control lines through a by-cohabitation protocol.

For the by-cohabitation components, we used the protocols previously described in [24, 25]. As described for the by-injection protocol, naïve and unselected hatchery-produced oysters were injected with a specific pathogen and then transferred into tanks for 24 h. Then, they were placed in contact with the naïve oysters of the selected and control lines to test their disease resistance. A ratio of 10 g of injected oysters (with the shell) per 10 L of sea water was used for all of the experiments. A dead oyster was defined as a moribund animal that was unable to close its valve after 5 min out of the water.

### Trial 1: experimental infection by cohabitation between oysters injected with OsHV-1 and the selected and control lines

An experimental infection with OsHV-1 was performed in April 2013 to verify the higher resistance to OsHV-1 infection of the selected lines AS and BS compared with the control lines AC and BC. The oysters were 13 months old, and the mean individual weight was 22 g (Table 1). The AS, BS, AC and BC lines were evaluated throughout the cohabitation with oysters injected with a viral suspension as described above. For each line (AS, BS, AC and BC), four 5 L replicate tanks were used; each tank contained 10 oysters (Table 1). For three replicates, 4 oysters injected with the viral suspension were added to each tank for 48 h. In the fourth tank, 4 oysters injected with SASW were added for 48 h. The mortality was recorded daily for 11 days.

**Table 1:** **Summary of the trials and sets to evaluate OsHV-1 and** *V. aestuarianus* **susceptibility.**

Trial	1	2	3	3	3	3	3	4	4
Set			1	2	3	4	5^a^	1^b^	2^c^
Pathogen	OsHV-1	*Vibrio aestuarianus*	*Vibrio aestuarianus*	*Vibrio aestuarianus*	*Vibrio aestuarianus*	*Vibrio aestuarianus*	*Vibrio aestuarianus*	*Vibrio aestuarianus*	*Vibrio aestuarianus*
Infection protocol	Cohabitation	Injection	Cohabitation	Cohabitation	Cohabitation	Cohabitation	Cohabitation	Cohabitation	Cohabitation
Number of exposure	1st infection	1st infection	1st infection	1st infection	1st infection	1st infection	1st infection	2nd infection	3rd infection
Date of challenge	Apr 2013	Jan 2013	Feb 2013	Mar 2013	Mar 2013	May 2014	Nov 2014	May 2014	Nov 2014
Age (months)	13	10	11	12	13	26	32	26	32
Individual weight (g)	22	22	22	22	22	120	100	100	170
Stage	Juvenile	Juvenile	Juvenile	Juvenile	Juvenile	Adult	Adult	Adult	Adult
Number of tanks^f^	12	48	3	3	8	2	2	1	1
Tank volume (L)	5	5	150	150	10	150	150	150	150
Lines tank^−1^	1	1	4	4	1	4	4	4	3^d^
Oysters line-1 tank^−1^	10	10	25	25	15–20	24–32	28–33	1–88^e^	1–30^e^
OsHV-1 detection in moribunds	Yes	No	No	No	No	No	No	No	No
*V. aestuarianus* detection in moribunds	No	Yes	Yes	Yes	Yes	Yes	Yes	Yes	Yes

For all trials, two unselected and control lines (AC and BC) and two lines selected for their higher resistance to OsHV-1 (AS and BS) were evaluated under controlled conditions

^a^Oysters used were survivors from field testing

^b^Oysters used were survivors from sets 1 to 3 of trial 3

^c^Oysters used were survivors from set 1 of trial 4

^d^One of the control line had 100% mortality before the trial

^e^Selected lines had at least 28 and 12 oysters in sets 4 and 5, respectively, whereas the control lines had less than 5 oysters

^f^The number indicated is without the control tanks

### Trial 2: experimental infection by injection of the selected and control lines with *V. aestuarianus*

The design of this trial consisted of intramuscular injection of the oysters with suspensions with different bacterial concentrations. The bacterial concentration was evaluated spectrometrically at 600 nm and adjusted to an optical density (OD) = 1; then, the suspension was serially diluted to obtain theoretical ODs of 0.0002, 0.002, 0.02 and 0.2, corresponding to 10^4^, 10^5^, 10^6^ and 10^7^ bacteria per mL, respectively. The bacterial concentration and purity were verified by plating. Three 5 L tanks were used for each OD and each line; each tank contained ten oysters injected with 50 µL of *V. aestuarianus* (500 CFU at OD 10^4^ and 0.5 million CFU at OD 10^7^). For each line, an additional tank was used as a control; this tank contained 10 oysters injected with SASW. Observations for mortality were performed daily for 6 days.

### Trial 3: primary infection by cohabitation between oysters injected with *V. aestuarianus* and the selected and control lines

Five sets of trials using a by-cohabitation protocol were used to better mimic natural infection. For each trial (with the exception of set 3), three 150 L tanks were used to challenge a larger number of larger animals at the same time. An additional tank was used for the controls, which consisted of oysters intramuscularly injected with SASW and placed as sources in contact with the four lines (Table 1). In each tank, the oysters of the four lines were randomly placed together in the same tank with 25 oysters per line with the shells individually tagged for identification. For set 3 of trial 3, two 10 L tanks were used per line; each tank contained approximately 15–20 oysters (Table 1).

The five sets of trial used to evaluate the resistance of the four lines AS, AC, BS and BC to *V. aestuarianus* are summarized in Table 1 and Figure 2:

- Three sets were performed in February and March 2013. The naïve oysters were 11–13 months old and weighed 22 g (Table 1), which corresponded to the juvenile stage according to the oyster industry. All oysters were always kept in our facilities, and no mortality was recorded.
- The fourth set was conducted during spring 2014. The oysters were 26 months old, and the mean individual weight was 120 g (Table 1), which corresponded to the adult stage. The oysters were always kept in our facilities, and no mortality was recorded.
- The fifth set was conducted during the fall of 2014 with oysters kept for 2 years in the field at Agnas (Charente Maritime, France). The control and selected oyster lines experienced 70 and 34% mortality, respectively. The oysters were 32 months old, and the mean individual weight was 100 g.

### Trial 4: successive infections by cohabitation of oysters injected with *V. aestuarianus* and the selected and control lines

All oysters that survived sets 1–3 in trial 3 were again challenged in trial 4 with two additional successive challenges in May 2014 and November 2014 (Figure 2). Between trial 3 and the first set in trial 4 and between the two sets of trial 4, the oysters were kept at the Ifremer facilities in La Tremblade. All effluent from the holding facilities was treated with chlorine. The occurrence of mortality was also recorded during these periods. In set 1 of trial 4, the oysters were 26 months old with an average weight of 100 g, whereas in set 2 of trial 4 the oysters were 32 months old with an average weight of 170 g (Table 1).

### Detection of OsHV-1 and *V. aestuarianus* DNA

For all of the trials, moribund oysters from the selected and control lines were sampled for the detection of OsHV-1 and *V. aestuarianus* DNA. Total DNA was extracted from tissue fragments (mantle + gills) using the QIAgen (Hilden, Germany) QIAamp tissue mini kit combined with the QIAcube automated system according to the manufacturer’s protocol. The total DNA amount was adjusted to 5 ng/µL following Nanodrop (Thermo Scientific) measurement.

A real-time PCR assay was conducted on the MX3000 and MX3005 Thermocyclers (Agilent) using the Brilliant III Ultrafast kit (Stratagene). Each reaction was run in duplicate in a final volume of 20 µL containing the DNA sample (5 µL at a 5 ng/µL concentration), 200 nM of each primer (for OsHV-1, DPF 5′ ATT GAT GATGTG GAT AAT CTG TG 3′ and DPR 5′ GGT AAA TAC CAT TGG TCT TGTTCC 3′ [26] and for *V. aestuarianus*, DNAj-F 5′ GTATGAAATTTTAACTGACCCACAA3′ and DNAj-R 5′ CAATTTCTTTCGAACAACCAC 3′ [27]) and 200 nM of an oligonucleotide probe (for *V. aestuarianus* DNAj, probe 5′ TGGTAGCGCAGACTTCGGCGAC). The real-time PCR cycling conditions were as follows: 3 min at 95 °C, followed by 40 cycles of amplification at 95 °C for 5 s and 60 °C for 20 s. For OsHV-1 DNA quantification, melting curves were also plotted (55-95 °C) to ensure that a single PCR product was amplified for each set of primers. Negative controls (without DNA) were included.

### Evaluation of the immune status of the selected and control lines

The immune statuses of the oyster lines were evaluated based on the expression of immune-related genes in the AS, AC, BS and BC lines prior to trial 3 set 1 at 12 months and trial 3 set 5 at 32 months. Immune-related genes were selected based on previous studies showing their transcriptomic regulation following vibrio challenge or between oyster lines selected for their resistance/sensitivity to in situ mortality [16, 18]. Oysters were removed from their shells, and the whole soft body was immediately plunged into liquid nitrogen. Then, the oysters were pulverized in groups (three groups of 10 oysters per oyster line) with a Mixer Mill MM 400 (Retsch) under liquid nitrogen conditions. The frozen oyster powder was stored at −80 °C prior to RNA extraction for gene expression analysis.

RNA extraction from the frozen oyster powder was performed with the TRIzol Reagent (Invitrogen) according to the manufacturer’s instructions. Briefly, 100 mg of oyster powder was homogenized in 1 mL of TRIzol by vortexing for 1 h at 4 °C. Next, the RNA samples were treated with 5 U of DNase I (Invitrogen) to eliminate DNA contamination according to the manufacturer’s instructions, followed by RNA precipitation to eliminate the degraded DNA (with 100% isopropyl alcohol and 3 M Na-acetate). Then, the RNA samples were dissolved in 50 µL of RNase-free water and quantified using a *NanoDrop* spectrophotometer (Thermo Scientific). The integrity of the total RNA was verified using 1.5% agarose gel electrophoresis. Finally, total RNA was reverse transcribed using the Moloney Murine Leukemia Virus Reverse Transcriptase (MMLV-RT) according to the manufacturer’s instructions (Invitrogen).

qPCR assays were performed on the Light-Cycler 480 System (Roche) in a final volume of 5 µL containing 1× Light-Cycler 480 master mix, 0.5 μM of each primer and 1 μL of cDNA diluted 1/8 in sterile ultra-pure water. The primer pairs used to amplify the 31 immune-related genes are listed in Table 2. Primer pair efficiencies (E) were calculated by five serial dilutions of pooled cDNA ranging from 1/2 to 1/64 in sterile ultra-pure water using the slopes provided by the LightCycler software according to the equation: E = 10^[−1/slope]^. The qPCR program was composed of an initial denaturation step of 15 min at 95 °C, followed by amplification of the target cDNA (35 cycles of denaturation at 95 °C for 10 s, annealing at 57 °C for 20 s and extension at 72 °C for 25 s with fluorescence detection). Relative expression levels of the immune-related genes were calculated with the method described by Pfaffl [28] and normalized using the mean of values of three constitutively expressed genes (*Cg*-*EF1* [GenBank AB122066], *Cg*-*RPL40* [GenBank FP004478] and *Cg*-*RPS6* [GenBank HS119070]).

**Table 2:** **Primers and functional categories of the analyzed immune-related genes.**

Gene number	Functional category	Name	Sense primer	AntiSense primer
72	Immune response	Metallothionein a	CAGCTCACACAGTCCCTTC	CATGTACAGTTACACGATGC
122	Immune response	Universal stress protein	TTGAGGTTTCCGTGAACGAG	AACAATCACCGGAACTGACG
130	Immune response	Interferon-induced protein 44	AAGATCCAACGATGAAAGAC	TTGTCGACATCACTACAAAC
163	Immune response	Big defensin	TTCGCCTGCTTCCATACTGG	GTCATGGTCACTCCTTATTC
189	Immune response	C-type lectin 2 like protein	GTCATCTGACCACAATTACAG	TCGATAGCAGCATTCCAGAG
220	Immune response	MyD88 adaptor	AGGTACCGGCTGTGATACGA	TTCAAACGCCACCAAGACTG
234	Immune response	Tumor necrosis factor ligand superfamily	GGATACGCAAGAGGAACTGC	TGGACATTAACGACACGCGC
293	Immune response	Interleukin 17	ACTGAGGCTCGATGCAAGTG	AGCCTTCTTGCTTCATGTGG
300	Immune response	Heat shock protein 70	GCATGTGAGCGAGCAAAACG	TGGCAGCTTGAACAGCAGC
303	Immune response	Galectin	ACGAAACGCTCTGATTGGTG	TTAGTGGCATGGTAGGTCTG
304	Immune response	L-rhamnose-binding lectin	AGATGATTGTGAAAGCAGCGA	ACTGTAGCGGTCATGCTCTG
306	Immune response	Proline rich protein	CACCATGTTCTCTCGGAGGA	GTCTGCAATGTTAACCCTCAG
307	Immune response	Hemocyte defensin	GTTGTAGAGCGGGCTACTGTG	CTTGGTCAGATTCAGACTGG
351	Immune response	Metallothionein b	GGAACTGTAGCTGTGGAGAC	CCTTCTTACAGCAGCAGTCG
399	Immune response	Metallothionein c	ATGTCCGATCATTGTTCCTG	ACAGGTTTCTGGTCCGTGAC
8	Cellular differentiation	Angiopoietin-1 receptor a	TGACGTGCTCGGCAACATGC	CATTGTGTCCCCGTGAAGCC
312	Cellular differentiation	Angiopoietin-1 receptor b	CGAAATCGTCTTACGAACGC	GTTAGCAAGATCCCGTTGAG
324	Cellular differentiation	Early growth response protein	CTACCTCCACAAGCGACATG	ACGTCGTTACTATGTGAGGG
216	Cellular differentiation	Placental protein 11	GCCAGATTTACCTGGAATGG	ATGCGGTGTAGATAGCGATG
375	Cellular differentiation	GTP-binding protein Di-Ras2	TTGGGCGTACAGTGACAACC	TCTCTGTTTCCTCGTGAACC
396	Cytoskeleton reorganization	Acyl-CoA desaturase	AGATGCAGACCCACACAACG	GCGTTCCAAAGTGATTCTCC
401	Cytoskeleton reorganization	Neurotrypsin	AAACAATGCAAGGGAGAAGC	CTATTGTCAGCACAATCTGG
441	Cytoskeleton reorganization	Major vault protein	TTCAAGAGTCAAGTGGATGC	ACCATTGGCGGTATTGAAGG
439	Cytoskeleton reorganization	Myosin essential light chain	TACATAACGGGTCATGAGAC	CAACACTGGATTACCACCTG
348	Cytoskeleton reorganization	Calcineurin subunit B isoform 1	ACGGTGTATTCCTTGTGTCC	TCTTCTGTACATGCAAGTGG
284	Cell adhesion-communication	Integrin beta-PS	CCCACCTAGTGCCAGTCAAG	GAACTTTGACTTGTGTGACGT
420	Cell adhesion-communication	Hemagglutinin/amebocyte aggregation factor precursor	TCGTGAATGCTGAACACACC	TACACCTGTCCAAACCAAGG
283	Respiratory chain	Extracellular superoxide dismutase	AGAGGTGAATGCTACCAGG	AGGCCAAGAATTCCGTCTG
422	Respiratory chain	Glutathione transferase omega-1	TTGGACAGGTTACCACACAG	CAAACCAAGGCCATACCATG
378	Pro- and anti-apoptosis	Caspase 7	AGGGAGACAAGCGCCGTCAG	TCCTCATTTGCTCTTCGTTC
166	No hit	Unknown gene product 166	AAGTCGTATAGGAGCACAGG	GGCTGAGAACATAATCCTCC

### Statistical analyses

Survival was analyzed with the SAS 9 software using the GLIMMIX procedure by a logistic regression for binomial data. The general model for the first trial was:

$$Y_{i} = Logit\left( \pi \right) = \ln \frac{\pi }{1 - \pi } = \mu + stock_{i} + selection_{j} + stock_{i} *selection_{j} + \varepsilon$$

where *Y*_*i*_ was the survival probability, *µ* was the intercept, *stock*_*i*_ represented stock A or line B, and *selection*_*j*_ represented the level of selection for OsHV-1 resistance (selected or control).

For trial 2, the bacterial concentration factor and all interactions between the bacterial concentrations, the stock and the selection factors were added to the model.

For the first three sets of challenges in trial 3, the set factor and its interactions were added to the model. A similar model was also used for the last two sets of challenges in trial 4.

For the gene expression analysis, statistical analysis was performed using the STATISTICA software version 7.1 (StatSoft) using the Mann–Whitney U test (significant value: *p* < 0.05). Hierarchical clustering of the gene expression data was performed with the Multiple ArrayViewer software using the average linkage clustering with the Spearman Rank Correlation as the default distance metric.

## Results

### Trial 1: experimental infection by cohabitation between oysters injected with OsHV-1 and the selected and control lines

No mortality occurred in the control tanks for each line (AC, AS, BC and BS). The first mortality occurred on day 2, and a peak of mortality was observed on day 5. As expected, the selected oysters presented low mortality (3 and 7% for AS and BS, respectively), whereas the control oysters had significantly higher mortality (60 and 67% for AC and BC, respectively, *p* < 0.001). The mean mortality was 32 and 37% for stocks A and B, respectively. The mortality between these stocks was not significantly different. Moreover, no difference was observed for the interaction between the stocks and the level of selection for resistance to OsHV-1 infection (*p* > 0.05). High amounts of OsHV-1 DNA was detected in all of the moribund oyster samples analyzed (*n* = 31).

### Trial 2: experimental infection by injection of the selected and control lines with *V. aestuarianus*

**Figure 3:**
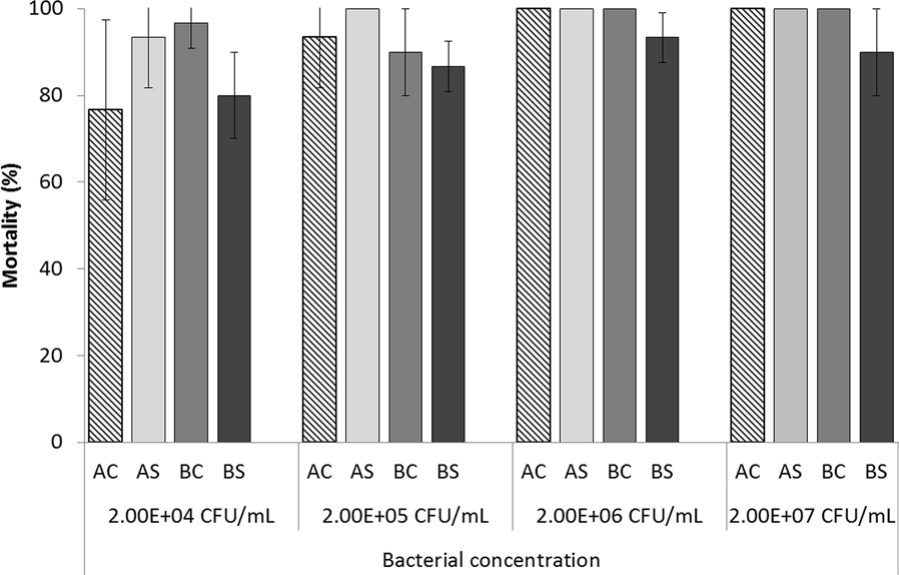
**Mean mortality (sd between tanks) in Trial 2.** Oyster lines were challenged at the juvenile stage via intramuscular injection of *V. aestuarianus* for the control (AC and BC) and selected lines (AS and BS) at different bacterial concentrations.

No mortality was observed for oysters injected with SASW in the control tanks. The mortality rates of each line at each injected dose are shown in Figure 3. Very high mortality ranging from 77 to 100% was observed for all lines regardless of the infectious dose. None of the factors were significant with the exception of the bacterial concentration factor (*p* = 0.0003) and the interaction between the stock and level of selection (*p* = 0.0005) (Table 3). At the stock level, the selected line exhibited lower mortality compared to the control line for stock B at each bacterial concentration; the opposite effect was observed for stock A at the two lowest concentrations (Figure 3). The mortality at the lowest bacterial concentration was significantly lower compared to the morality at the other concentrations (*p* < 0.0001).

**Table 3:** **Logit analysis of mortality in Trial 2.**

Effect	DF	F	*P*
Stock	1	2.42	0.1207
Selection	1	0.6	0.4374
Bacterial concentration	3	6.29	0.0003
Stock × selection	1	12.23	0.0005
Stock × bacterial concentration	3	1.31	0.2708
Selection × bacterial concentration	3	0.5	0.6801
Stock × selection × bacterial concentration	3	2.06	0.1042

Oyster lines were challenged at the juvenile stage for primary infection by injection of *V. aestuarianus* for the control and selected lines of both stocks and at different doses

### Trial 3: primary infection by cohabitation between oysters injected with *V. aestuarianus* and the selected and control lines at the juvenile and adult stages

#### Primary exposure with the *V. aestuarianus* cohabitation protocol at the juvenile stage (sets 1 to 3)

**Figure 4:**
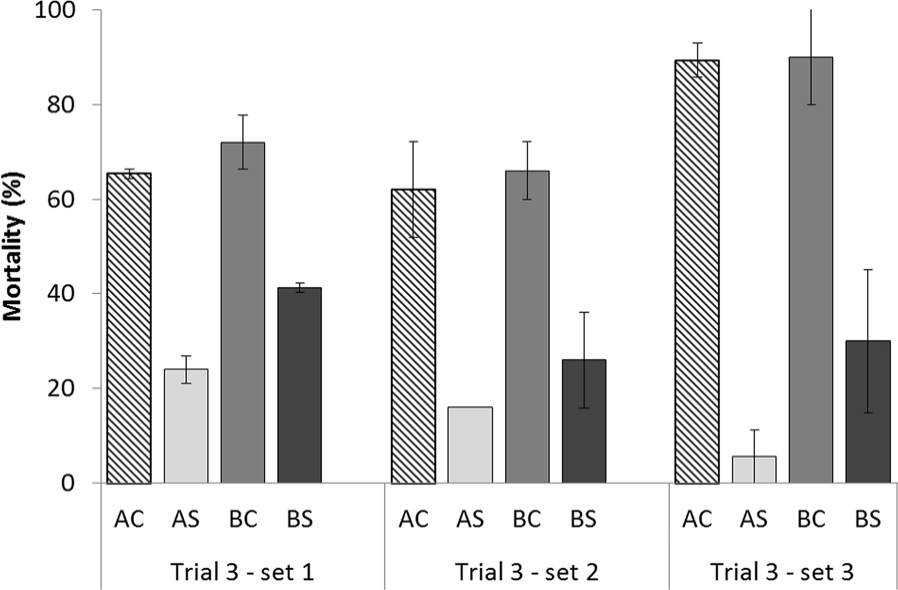
**Mean mortality (sd between tanks) in Trial 3 sets 1 to 3.** Oyster lines were challenged at the juvenile stage via cohabitation challenges between oysters injected with *V. aestuarianus* and healthy juveniles of the control (AC and BC) and selected lines (AS and BS).

No mortality occurred in the control tanks of each set of trial 3. All moribund oysters sampled were positive for *V. aestuarianus* DNA (*n* = 45). The mortality of each line at the endpoint for the first three sets of trial 3 (corresponding to the juvenile stage) is presented in Figure 4. In contrast to the by-injection protocol, higher variability in mortality was observed among the lines, with a range from 6 to 90%. A significant interaction was found between the sets and selection (*p* = 0.0008) (Table 4); this interaction was explained at the selection level, with higher mortality observed in set 1 compared to set 3 for the selected lines and the highest mortality observed in the control lines in set 3 (Figure 4). The mean mortalities of the three sets were 14 and 33% for the AS and BS lines, respectively; this mortality was significantly lower than the mortality observed for the control lines (71 and 80% for AC and BC, respectively, *p* < 0.0001) (Table 4). To a much lesser extent, stock A had significantly lower mortality (43%) than stock B (53%) (*p* = 0.0065).

**Table 4:** **Logit analysis of mortality in Trial 3 sets 1 to 3.**

Effect	DF	F	*P*
Set	2	2.41	0.0909
Stock	1	7.46	0.0065
Selection	1	107.19	<.0001
Set × stock	2	0.43	0.6518
Set × selection	2	7.25	0.0008
Stock × selection	1	3.81	0.0515
Set × stock × selection	2	0.64	0.8576

Oyster lines were challenged at the juvenile stage for a primary infection throughout 3 sets of cohabitation between oysters injected with *V. aestuarianus* and the control and selected lines of both stocks

#### Primary exposure with the *V. aestuarianus* cohabitation protocol at the adult stage (sets 4 and 5)

**Figure 5:**
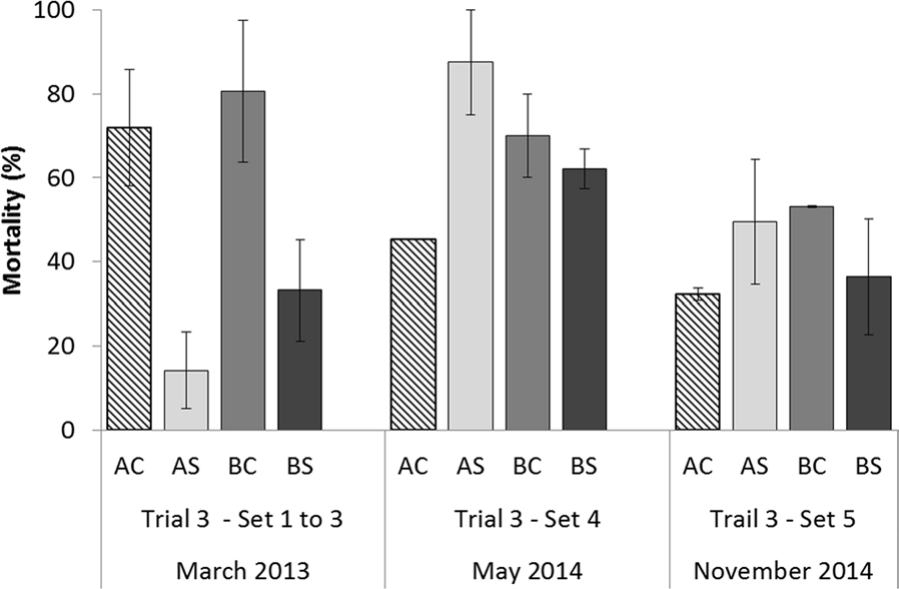
**Mean mortality (sd within** ***l) in Trial 3 sets 4 and 5.*** Oyster lines were challenged at the juvenile stage for the three first sets of trial 3 and at the adult stage for sets 4 and 5 of trial 3 via cohabitation challenges between oysters injected with *V. aestuarianus* and healthy juveniles of the control (AC and BC) and selected lines (AS and BS).

For the fourth set of trial 3, the mortality was 87, 45, 62 and 70% for the AS, AC, BS and BC lines, respectively; the mortality decreased to 49, 32, 36 and 53%, respectively, in the fifth set of challenges in trial 3 (Figure 5). None of the factors were significant with the exception of a significantly lower mortality in the fifth set compared to the fourth set (*p* = 0.0002) and a significant interaction between the stocks and level of selection (*p* = 0.0012) (Table 5). At the stock level, the stock A control line had significantly lower mortality than the selected line, whereas the opposite trend was observed for stock B (Figure 5).

**Table 5:** **Logit analysis of mortality in Trial 3 set 4 and 5.**

Effect	DF	F	*P*
Set	1	13.85	0.0002
Stock	1	0.00	0.9557
Selection	1	2.23	0.1368
Set × stock	1	0.40	0.5278
Set × selection	1	2.39	0.1233
Stock × selection	1	10.72	0.0012
Set × stock × selection	1	0.82	0.3648

Oyster lines were challenged at the adult stage for a primary infection throughout 2 sets of cohabitations between oysters injected with *V. aestuarianus* and the control and selected lines of both stocks

### Expression levels of immune-related genes can discriminate oyster lines in terms of susceptibility/resistance to *V. aestuarianus* infection at the juvenile and adult stages

**Figure 6:**
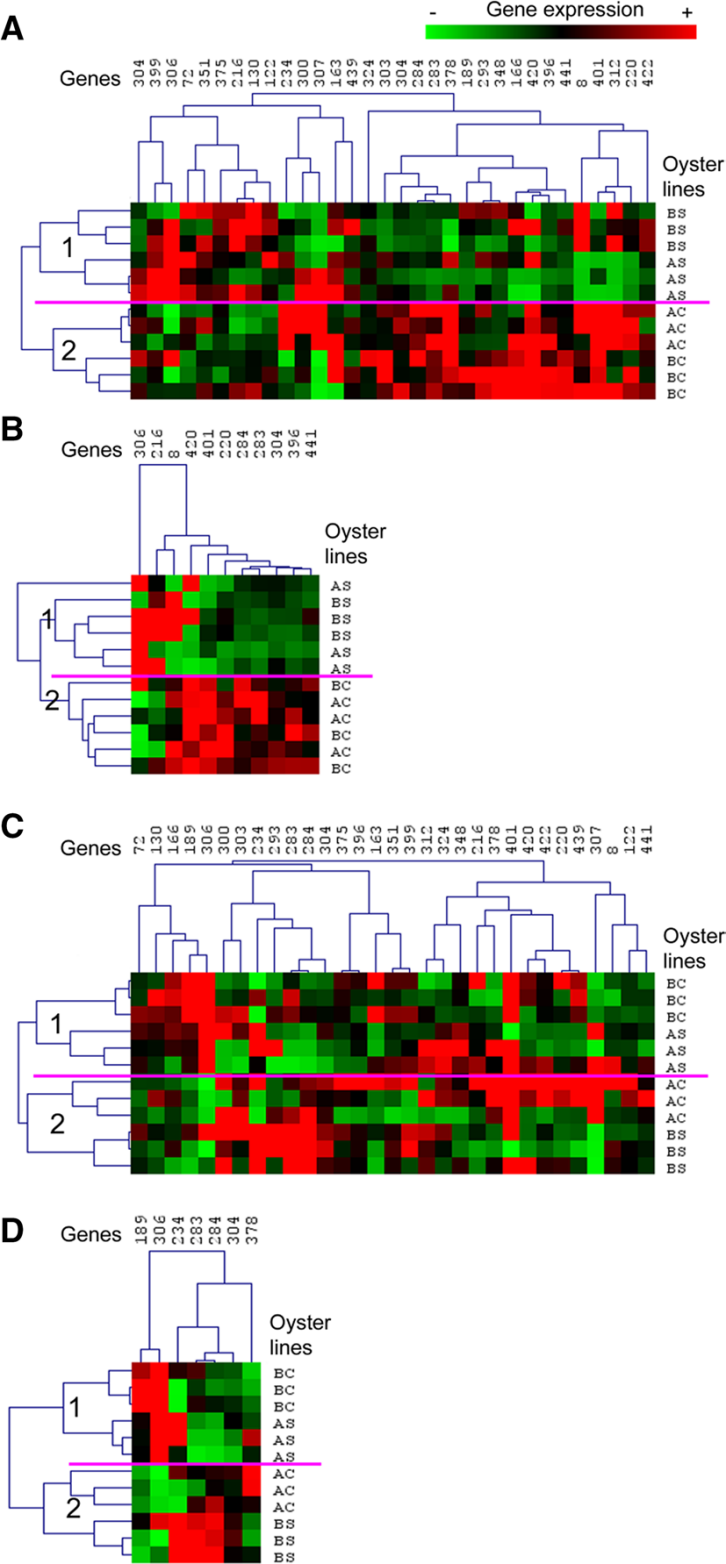
**Discrimination of oyster lines contrasted in term of susceptibility based on the expression levels of immune-related genes.** **A** Hierarchical clustering of the relative expression levels of 31 immune-related genes in non-stimulated oysters of the AC, AS, BC and BS lines (three groups of ten oysters per line) at 12 months of age. **B** Hierarchical clustering of the relative expression levels of the 11 differentially expressed genes in non-stimulated oysters of the AC, AS, BC and BS lines (three groups of ten oysters per line) at 12 months of age. **C** Hierarchical clustering of the relative expression levels of 31 immune-related genes in non-stimulated oysters of the AC, AS, BC and BS lines (three groups of ten oysters per line) at 32 months of age. **D** Hierarchical clustering of the relative expression levels of the 7 differentially expressed genes in non-stimulated oysters of the AC, AS, BC and BS lines (three groups of ten oysters per line) at 32 months of age. The intensity of the color (from green to red) indicates the magnitude of differential expression (see color scale at the bottom of the image). The dendrogram at the left of the figures indicates the relationship among samples from the oyster lines, whereas the dendrogram at the top of the figures indicates the relationship among the relative expression levels of the selected genes.

The hierarchical clustering of the 31 immune-related genes or only the differentially expressed genes (*p* < 0.05) could separate the oyster lines in terms of their resistance/sensitivity to bacterial infection at the two developmental stages analyzed (12 and 32 months, Figure 6). At 12 months of age, hierarchical clustering of the gene expression data separated the oyster lines into two major clusters of conditions: the first cluster included the AS and BS lines, while the second cluster included the AC and BC lines (Figure 6A). At 32 months of age, two major clusters of conditions were found that were similar to those observed for the 12 month old oysters, but the oyster lines did not separate in the same manner (Figure 6B): the first cluster included the AC and BS lines, whereas the second cluster included the AS and BC lines. Interestingly, these discriminations of the oyster lines were in accordance with the resistance/sensitivity to infection of the lines at 12 and 32 months of age.

In the juveniles, 11 genes showed differential gene expression patterns, while in the adults only 7 genes showed differential expression patterns. Among these differentially expressed genes, four genes were common to juveniles and adults (306, 283, 284 and 304). These four genes showed the same patterns of expression in the juveniles and adults according to their resistance/sensitivity to infection. Thus, genes expressed at higher levels in the susceptible lines (AC and BC) vs. the resistant lines (AS and BS) in juveniles also appeared to be expressed at higher levels in the susceptible lines (AC and BS) vs. the resistant lines (AS and BC) in adults (genes 283, 284 and 304). Likewise, the gene expressed at a higher level in the resistant lines (AS and BS) vs. the susceptible lines (AC and BC) in juveniles also appeared to be expressed at a higher level in the resistant lines (AS and BC) vs. the susceptible lines (AC and BS) in adults (gene 306). Other differentially expressed genes appeared to be associated with one developmental stage: 7 genes were found to be differentially expressed only in juveniles (216, 8, 420, 401, 220, 396 and 441), and 3 genes were found to be differentially expressed only in adults (189, 234 and 378).

### Trial 4: successive infections by cohabitation between oysters injected with *V. aestuarianus* and the selected and control lines

After the three first sets of bacterial challenges of trial 3, the final mortality was 14, 71, 33 and 80% for AS, AC, BS and BC, respectively. During the period between trials 3 and 4, the survivors were kept in a tank with filtered and UV-treated seawater enriched with microalgae. Although this period did not represent a disease challenge, the survivors still experienced significant mortality associated with the detection of *V. aestuarianus* DNA in the moribund oysters. Most of the mortality was observed in July 2013 after a spawning event. The mortality between trial 3 and the first set of challenges of trial 4 was 74, 90, 32 and 97% for AS, AC, BS and BC, respectively (Table 6); consequently, the cumulative mortality due to *V. aestuarianus* before trial 4 reached 78, 96, 54 and 99%, respectively. Although the control lines were tested in trial 4, the remaining oysters numbered less than 5. Therefore, the mortality was not compared with the selected lines. During the first set of challenges in trial 4, the mortality was 46 and 28% for AS and BS, respectively (Table 5). No significant difference in mortality (<5%) was reported between the two sets of challenges in trial 4. Finally, the survivors exhibited some mortality, with 60 and 38% mortality for AS and BS, respectively (Table 6). The cumulative mortality after three successive challenges with *V. aestuarianus* in trials 3 and 4 (including the mortality between trials) was 96, 99, 84 and 100% for AS, AC, BS and BC, respectively (Table 6).

**Table 6:** **Mortality rates per line during three successive challenges.**

	AS	AC	BS	BC
Initial number of oysters	160	153	165	155
Mean mortality of sets 1 to 3 of trial 3 (Feb to Mar 2013) (%)	14	71	33	80
Mortality between trial 3 and the first set of trial 4 (%)	74	90	32	97
First set of trial 4 in Mar 2014 (%)	46	*40*	28	*100*
Mortality between the two sets of trial 4 (%)	<5	<*5*	<5	–
Second set of trial 4 in Nov 2014 (%)	60	*0*	38	–
Final oysters number remaining after three successive challenges	6	*1*	26	0
Cumulated mortality after three successive challenges (%)	96	99	84	100

Cumulative mortality by cohabitation between oysters injected with *V. aestuarianus* and the control (AC and BC) and selected lines (AS and BS) are shown from sets 1, 2, and 3 of trial 3 and sets 1 and 2 of trial 4

In italics, the number of oysters was less than 5

## Discussion

While mortality related to OsHV-1 and *V. aestuarianus* was reported in *C. gigas* in France prior to 2008 [27, 29], their impact on French oyster production became predominant due to recurrent and intense mortality in spat and adult oysters. While selective breeding to enhance resistance to OsHV-1 infection in *C. gigas* has been recently demonstrated [11, 15], this demonstration is under investigation for *V. aestuarianus*. Nevertheless, this study aimed to elucidate whether selective pressure exerted by viral infections in the field could impact the susceptibility of *C. gigas* to bacterial infection.

In trial 1, the selected lines of both stocks exhibited much lower mortality (AS 3% and BS 7%) than the control lines (AC 60% and BC 67%). Although higher mortality was observed in the field evaluation (50 and 35% for AS and BS, respectively, and 91 and 94% for AC and BC, respectively [11]), our result was consistent with the field evaluation and supported that selection to enhance survival in *C. gigas* spat was effective for herpes virus infection. The lower mortality observed in our study could be explained by the use of larger (20 g versus <7 g) and older (13 months old versus <5 months old) oysters (juvenile versus spat) because OsHV-1 resistance increased with age and size [21]. However, the most important information from trial 1 was the confirmation that AS and BS had higher resistance to OsHV-1 infection than AC and BC before their evaluation of exposure to *V. aestuarianus*.

In trial 2, the mortality of three of the lines reached 100%. The mortality for BS was >90% at higher bacterial concentrations corresponding to 5 × 10^4^ and 5 × 10^5^ CFU per oyster (Figure 3). Although the mortality was slightly lower at the lowest bacterial concentration (corresponding to 500 CFU per oyster and ranging from 76 to 96%; Figure 3), this finding confirmed that strain 02/041 was a highly virulent strain in *C. gigas* juveniles (20 g); this finding was recently demonstrated in spat weighing 1.5 g that exhibited high mortality (>75%) at doses of 10^2^ and 10^7^ CFU per spat [30]. Consequently, selective breeding to enhance higher resistance to OsHV-1 at the spat stage but does not confer higher resistance to *V. aestuarianus* infection at the juvenile stage. Injection of the bacteria directly into the adductor muscle may bypass the oysters’ natural barriers to infection by *V. aestuarianus*. Due to the high mortality observed for all lines when the injection method is used, the cohabitation method should be applied to evaluate the resistance to *V. aestuarianus* infection of the control and the selected lines because transmission of the bacteria between oysters is what is expected to occur under natural conditions.

The three sets of trial 3 were performed at the juvenile stage, and all exhibited the same mortality patterns. The main finding revealed that the selected lines AS and BS had lower mortality than the control lines AC and BC (Figure 4). This result suggested that selection to increase survival at the spat stage in the field was also efficient in enhancing dual resistance to OsHV-1 infection and *V. aestuarianus* infection at the juvenile stage. A similar result was observed in *Crassostrea virginica* for dual resistance to *Haplosporidium nelsoni* and *Perkinsus marinus* [31], but most studies revealed that breeding for higher resistance to a disease did not confer a higher resistance to another disease [32].

Controversially, this pattern was not found at the adult stage, where much higher mortality was observed for both of the selected lines (particularly the AS line). Indeed, line AS exhibited 87% mortality in set 4 of trial 3, whereas the control line AC had lower mortality (45%) (Figure 5). Additionally, while the AS line had higher mortality in adults than in juveniles, the AC line had lower mortality at the adult stage (45%) than at the spat stage (71%) (Figure 5). This same pattern was observed for BS and BC to a lesser extent, except that BS had lower mortality than BC at the adult stage (Figure 5). A similar pattern was observed in set 5 of trial 3, although the mortality was lower than the mortality recorded in set 4 of trial 3. For set 5 of trial 3, it is important to note that the oysters were survivors of field mortality events that could have been related to OsHV-1 and/or *V. aestuarianus* and/or other pathogens. Consequently, the survivors used in set 5 were likely to be genetically more resistant than the naïve oysters used in set 4, as demonstrated for the summer mortality phenomenon in *C. gigas* [33]. Another hypothesis could be related to the reproductive status. Sets 4 and 5 of trial 3 occurred in May and November of 2014, which represented the pre- and post-spawning periods, respectively. Previous experiments have shown that the active gametogenesis period corresponds to higher susceptibility to vibriosis in mollusks [34–37]. Consequently, primary infection of *C. gigas* with *V. aestuarianus* by cohabitation showed a different mortality pattern according to the stage of development and the level of selection. Hence, evaluation of vibriosis resistance in *C. gigas* represents a complex interaction between the genotype and the stage of development, and therefore the size, reproductive status and age of the oysters as described for OsHV-1 in *C. gigas* [21, 33, 38]. Our study also revealed that selecting for resistance to OsHV-1 infection in spat did not confer either higher resistance or susceptibility to *V. aestuarianus* infection in adults, which was in agreement with similar studies in oyster species [39–41]. Experimental studies working on *V. aestuarianus* should replicate the oyster genotypes. The difference in mortality between the four lines used in our study also suggested a genetic basis for *V. aestuarianus* resistance in *C. gigas*, but this speculation required further investigation.

Our results showed high variability in the expression of selected immune-related genes that was dependent on the animal batch and age. This variability allowed the discrimination of oyster batches and correlated with their sensitivity to bacterial infection. Interestingly, expression of this set of immune-related genes was correlated with sensitivity to vibriosis rather than the genetic background. Because sensitivity to infection evolved depending on the oyster stage tested, the clustering of oyster batches also evolved in an age-dependent manner. At the juvenile stage, the lines selected for their resistance to OsHV-1 infection that presented low sensitivity to vibriosis were clearly discriminated from the control lines that presented higher sensitivity to *V. aestuarianus* infection. At the adult stage, selected line A and control line B showed intermediate sensitivity to vibriosis and were discriminated from control line A and selected line B with higher sensitivity to *V. aestuarianus* infection. Our results showed for the first time the possibility of using gene expression analysis to discriminate between oyster lines according to the resistance/susceptibility at two different developmental stages independent of the genetic background of the oyster lines. Specifically, four genes discriminated between the oyster lines according to their resistance/susceptibility.

These four genes are associated with different immune functions and suggest a complex discrimination of oyster lines through their immune status. The four genes able to discriminate oyster lines are related to antimicrobial functions (the proline rich peptide *Cg*-*prp* [42]), anti-oxidative responses (the extracellular superoxide dismutase *Cg*-*SOD* [43]), cell adhesion (the *Integrin beta*-*PS* [44]) and recognition molecules (the *L*-*rhamnose*-*binding lectin* [45]). These results show that it is now necessary to develop global transcriptomic approaches to clearly elucidate the transcriptomic basis of the resistance/susceptibility of oysters.

Finally, trial 4 was designed to test the effect of successive challenges using survivors of a previous challenge. The survivors of an initial exposure to *V. aestuarianus* still exhibited significant mortality in response to the same pathogenic agent at the second exposure. Consequently, the survivors were not genetically resistant, but they were either less susceptible during the previous exposures or the infection cohabitation did not allow equal expose of the oysters to the bacteria. Thus, a first contact with *V. aestuarianus* is not protective. This mortality pattern was also found in the abalone *Haliotis tuberculata* during two successive infections by the pathogen *Vibrio harveyi* [46]. Our result contrasted with the results obtained for the summer mortality phenomenon and OsHV-1, for which the survivors were selected for resistance and exhibited low mortality the following year [33, 38]. Between trials 3 and 4, mortality due to *V. aestuarianus* was mostly observed after a spawning event, thereby reinforcing the importance of the reproductive status on the resistance to the bacteria. Post-spawning oysters were much more susceptible to the disease, as demonstrated with the mortality event due to opportunistic *Vibrio* sp in *C. gigas* [34, 37, 47] and OsHV-1 in *C. gigas* that occurred a couple of days after spawning [48]. Otherwise, the cumulative mortality after three successive exposures to *V. aestuarianus* was very high for all lines (ranging from 84 to 100%) (Table 6). These mortality rates are extremely concerning for French oyster farmers, who cannot continue to remain feasible with this level of loss of *C. gigas* in their oyster stocks.

In conclusion, our study showed that: (1) cohabitation between injected oysters and healthy oysters seemed to be preferable for the genetic evaluation of *V. aestuarianus* resistance in *C. gigas* compared to intramuscular injection; (2) the mortality pattern for primary exposure to *V. aestuarianus* at the juvenile stage was similar to the pattern observed for OsHV-1 infection, with a higher resistance in selected oysters than control oysters, which suggested dual resistance at the juvenile stage; (3) differences in the mortality patterns were highlighted between juveniles and adults during primary infection, suggesting a complex interaction between the genotype and the stage of development for *Vibrio* sensitivity; and (4) selection of immune-related genes allowed for the discrimination of batches depending on their sensitivity to infection at the two stages tested rather than on their genotype. The differences in mortality among the lines also suggested a genetic basis for the resistance to *V. aestuarianus* infection. Similarly, selection to enhance OsHV-1 resistance did not confer increased susceptibility or resistance to *V. aestuarianus* infection. Therefore, to improve resistance to *V. aestuarianus* infection, a breeding program needs to use high intensities of selective pressure. Resistance to *V. aestuarianus* infection should be evaluated through successive exposures to the disease because the oysters remained susceptible to *V. aestuarianus* even if they survived one or several mortality outbreaks related to the disease. Breeding companies interested in enhancing *Vibrio* resistance should use oysters that were previously selected for resistance to OsHV-1 infection at the spat stage for broodstock. Then, these broodstocks should be evaluated at the adult stage to combine the resistance traits.
